# Completeness of Telehealth Interventions Reporting in Randomized Controlled Trials for Caregivers of People With Dementia: Systematic Review

**DOI:** 10.2196/53737

**Published:** 2025-01-20

**Authors:** Ling Zhu, Yurong Xing, Wenhui Xu, Hongfei Jia, Xiaoxiao Wang, Shiqing Liu, Yaping Ding

**Affiliations:** 1 Department of Basic and Community Nursing School of Nursing Nanjing Medical University NanJing China; 2 School of Nursing Nanjing Medical University NanJing China; 3 Nursing Department JiangSu Province Official Hospital NanJing China

**Keywords:** telehealth, intervention reporting, dementia, caregivers, Template for Intervention Description and Replication, TIDieR-Telehealth checklist

## Abstract

**Background:**

Telehealth interventions can effectively support caregivers of people with dementia by providing care and improving their health outcomes. However, to successfully translate research into clinical practice, the content and details of the interventions must be sufficiently reported in published papers.

**Objective:**

This study aims to evaluate the completeness of a telehealth intervention reporting in randomized controlled trials (RCTs) conducted for caregivers of people with dementia.

**Methods:**

A systematic search of relevant papers was conducted on July 26, 2023, in 9 electronic databases. RCTs of telehealth interventions for caregivers of people with dementia were included. Two independent researchers extracted the descriptive information and assessed the methodological quality (Cochrane risk of bias tool) and the completeness of reporting of the intervention by using the Template for Intervention Description and Replication (TIDieR)-Telehealth checklist, which consists of 12 items.

**Results:**

Thirty-eight eligible RCTs were included finally, and the overall quality of the studies was assessed as moderate. None of the studies completely reported all the TIDieR-Telehealth items. The most frequently reported items were the brief trial name (35/38, 92%), rationale (38/38, 100%), materials and procedures (35/38, 92%), and the modes of delivery (34/38, 90%). The least reported items were the type of location (0/38, 0%), modifications (4/38, 11%), and assessment and improvement of fidelity (9/38, 24%).

**Conclusions:**

Many details of the telehealth interventions in RCTs are reported incompletely. Greater adherence to the TIDieR-Telehealth checklist is essential for improving the reporting quality and for facilitating replicability, which has substantial implications for translation into clinical practice.

## Introduction

According to the World Alzheimer Report 2023, over 55 million people live with dementia worldwide, and this number is expected to reach 139 million by 2050 [1]. Alzheimer disease is ranked as the seventh leading cause of mortality and poses the highest burden of disease globally [2], and dementia care has become the focus of global health services [3]. To perform activities of daily living, most people with dementia require care from informal caregivers such as family members and friends or from formal caregivers [4]. Studies have shown that caregivers for people with dementia may experience emotional stress that probably results in adverse effects on the patients [5,6]. Reports indicate that these caregivers frequently experience symptoms such as depression, anxiety, and stress [7]. Currently, there are many interventions for supporting caregivers for dementia care. With the development of the society and the progress of technology, telehealth as an alternative medium for delivering health care has boomed.

Telehealth is defined as the use of medical information exchanged via electronic communication from one location to another [8]. Various types of telehealth interventions have been widely used in medical care, which have been shown to improve the quality of care and provide better outcomes for patients [9,10]. Caregivers of people with dementia require favorable interventions because of the complexity of dementia care, and telehealth can increase the opportunities for caregivers to receive interventions. Studies have shown that telehealth interventions have benefits for caregivers, such as improving their perceived competency, reducing their burden, and relieving their depression and stress [11,12].

Randomized controlled trials (RCTs) are currently recognized as the gold standard for evaluating clinical treatments and for generating high-quality evidence for the effectiveness and efficacy of interventions [13]. One challenge identified in the reporting of clinical trials is the quality of the descriptions of interventions [14]. The reproducibility of RCT results is fundamental to improving evidence-based care and patient outcomes, but insufficiently published descriptions of interventions will affect the reproducibility of trials, raising concerns about the validity and reliability of the findings [15].

Although the CONSORT (Consolidated Standards of Reporting Trials) statement offers general recommendations for intervention reporting, it lacks detailed guidance on critical intervention components such as the particular techniques and modes of delivery [12]. To address this gap, the Template for Intervention Description and Replication (TIDieR) checklist was published in 2014 to emphasize the importance of adequate reporting of interventions in clinical trials, enabling detailed descriptions of the intervention content for the reproducibility of trials [15,16]. In 2022, the TIDieR-Telehealth checklist, as an extension of the original TIDieR checklist, provided additional guidance specific to reporting of telehealth interventions with the goal of improving reporting quality and maximizing reproducibility and implementation in clinical trials [17].

Previous studies using the TIDieR checklist for assessment have shown incomplete intervention reporting [15,16,18], raising concerns about the comprehensiveness of telehealth intervention reports for dementia caregivers. To our knowledge, no study has yet examined the quality of reporting of interventions within dementia care interventions or identified which components are the most underreported. Therefore, the purpose of this study was to assess the completeness of telehealth intervention reporting in RCTs for caregivers of people with dementia.

## Methods

### Study Design

This review assesses the completeness of telehealth intervention reporting in RCTs for caregivers of people with dementia by using the TIDieR-Telehealth checklist.

### Search Strategy

Related papers were retrieved from PubMed, Embase, Cochrane Library, Web of Science, CINAHL, China National Knowledge Infrastructure, Chinese Biomedical Literature database, Wanfang database, and VIP Chinese Science and Technology Periodicals Full-Text database, without any restriction on the date of publication (from inception to July 26, 2023). Only RCTs published in English or Chinese were included. The search strategy can be found in Multimedia Appendix 1.

### Inclusion Criteria

The criteria for the inclusion of the published papers were as follows: (1) participants were caregivers providing care for people with dementia at any stage (formal or informal caregivers); (2) telehealth interventions involved any treatments that were delivered remotely via text messaging, videoconferencing, audio-only communication, mobile apps, and other telecommunication tools [17]; (3) the control group received usual care, standard care, waiting list, face-to-face, or any other nontelehealth interventions; (4) all eligible studies were analyzed for completeness of reporting of the intervention, regardless of the outcomes; and (5) only RCTs.

### Study Selection

Two independent researchers, systematically trained in evidence-based research, screened the titles and abstracts of the selected papers by using the inclusion criteria to identify potentially eligible studies. Full texts were obtained and again screened for final inclusion. Disagreements were resolved by a third researcher.

### Data Extraction

Two independent researchers extracted information regarding the characteristics, methodological quality, and completeness of reporting of the intervention. A third researcher resolved disagreements arising at any stage. Regarding the study characteristics, we extracted the following data: first author, year of publication, sample size, age and gender of included participants, severity of dementia, intervention characteristics, outcomes and measurement tools, assessment times, risk of bias, and completeness of reporting.

### Risk of Bias Assessment

The Cochrane Risk of Bias tool [19] was used to assess the methodological quality of the eligible trials on random sequence generation, allocation concealment, blinding of participants and personnel, blinding outcome assessment, incomplete outcome data, selective reporting, and other biases. Each item was rated as low risk, high risk, or unclear risk. In addition, the overall risk of bias of the study was classified as A (low risk in all domains), B (low risk in some domains), or C (no low-risk domains) [20]. Due to the high risk of bias and low quality of grade C studies, those studies were not included [20-22].

### Completeness of Reporting Assessment

The completeness of reporting was extracted using the TIDieR-Telehealth checklist, which has 12 items such as brief name, why, what (materials and procedures), who provided, how, where, when, and how much, tailoring, modifications, and how well (planned, actual). Considering that there are multiple aspects included in the items, each item was scored on a 3-point scale: 1 if all the criteria were met, 0.5 if some criteria were included, and 0 if no criteria were met or mentioned. The full score of each study was 12. It should be noted that item 10 was considered adequately reported if there was explicit mention of modifications during the study and was considered not adequately reported if no explicit mention of modifications was made during the study.

### Data Analysis

We performed a descriptive analysis to describe the included trials, the methodological quality, and the reporting of the interventions. Overall scores were calculated on the completeness of reporting for each study, and numbers and percentages were used to summarize the adherence to each item of the checklist for all the included trials. Additionally, we used a histogram to represent the number of trials with different scores on each item. The methodological quality of the eligible trials was shown by a risk-of-bias summary graph created in Review Manager version 5.4.

## Results

### Study Selection

**Figure 1:**
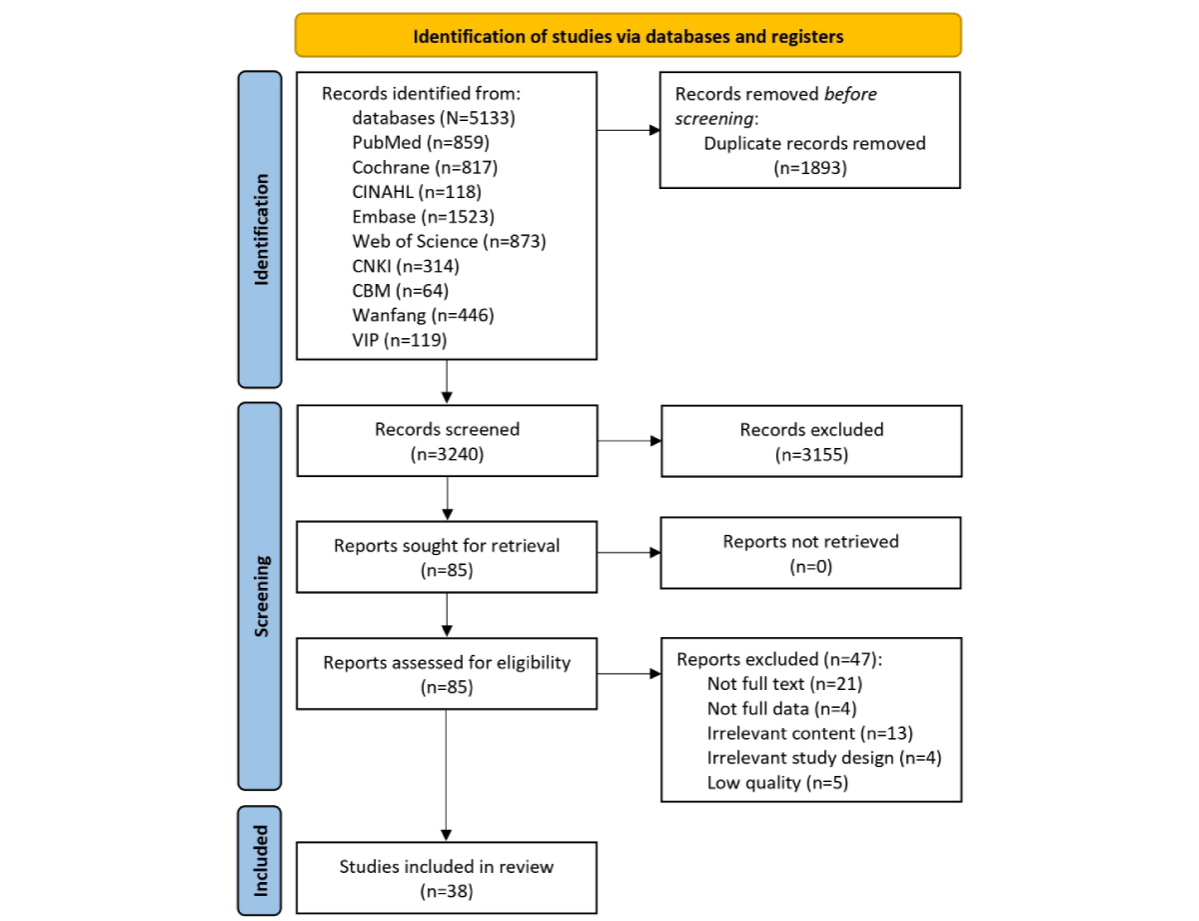
Flow diagram for the screening of studies. CBM: Chinese Biomedical literature; CNKI: China National Knowledge Infrastructure.

A total of 5133 results were obtained after the preliminary screening. After excluding 1893 duplicate studies and 3155 irrelevant studies that did not meet the criteria (as shown by their titles and abstracts), 38 studies were included after full-text review (Multimedia Appendix 2). The literature screening process and results are shown in Figure 1.

### Description of the Included Studies

The characteristics of the eligible RCTs are described in Multimedia Appendix 3 [23-60]. Considering the recent publication of the telehealth checklist, our study mainly conducted a retrospective evaluation of the completeness of reporting in the relevant papers. All 38 papers included in this review were published from 2003 to 2023 in 12 countries, namely, the United States (n=16) [32-40,43-45,48,55,56,58], Germany (n=6) [25,30,46,47,54,57], China (n=4) [23,42,52,59], Canada (n=2) [31,60], Spain (n=2) [49,50], Netherlands (n=2) [26,27], Belgium (n=1) [51], France (n=1) [28], Greece (n=1) [41], Portugal (n=1) [53], India (n=1) [24], and Italy (n=1) [29]. The total sample size was 4001 (range 14-273), with an average age range of 37-72 years. Seven studies [25,29,32,33,35,41,55] did not specify the proportion of females, while the other studies [23,24,26-28,30,31,34,36-40,42-54,56-60] included 2676 (79.4%) females. All the trial interventions were delivered by the internet or telephone, with 15 studies [23,25,29,30,35,42,44,45,47,51,54,55,57-59] using the telephone to provide education or support to caregivers. Only 17 of the 23 studies that conducted interventions through the internet mentioned specific modalities of the intervention, of which 13 [24,27,28,31,36,37,40,41,49,50,52,53,60] provided caregivers with knowledge, skills, and support through a web page or app, and 3 [32,33,43] studies provided support via a computer-telephone integration system. One study [34] provided a monitoring system for caregivers.

### Methodological Quality

**Figure 2:**
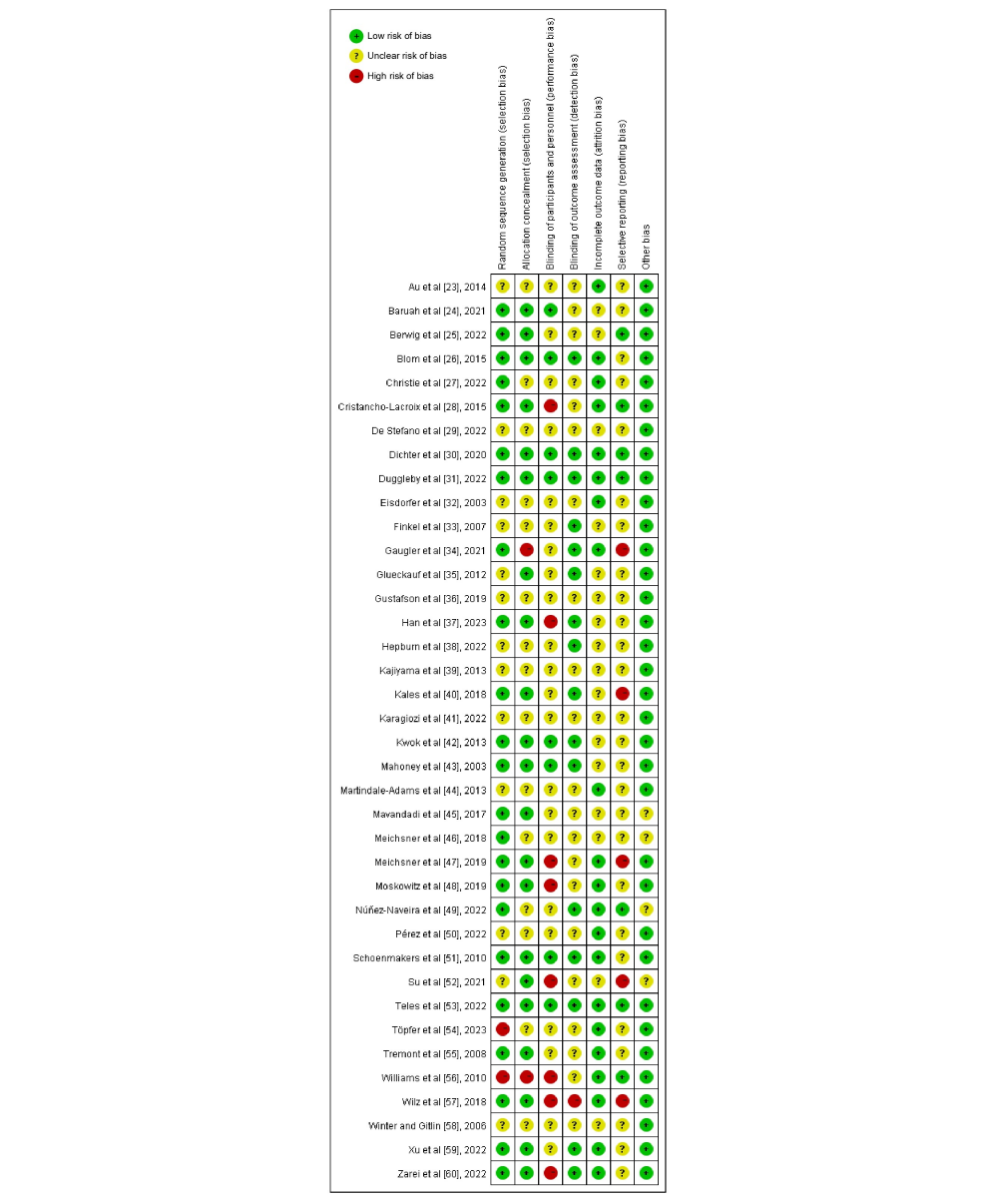
Risk of bias graph.

The risks of bias for the included studies is shown in Figure 2 [23-60]. According to the risk of bias assessed by the Cochrane handbook, 38 papers were qualified, with 3 studies graded as A [30,31,53] and 35 as B. In general, the methodological quality of these RCTs was moderate. Specifically, 23 studies [24-28,30,31,34,37,40,42,43,45-49,51,53,55,57,59,60] described the generation of random sequences. Allocation concealment was not reported in 15 papers [23,27,29,32,33,36,38,39, 41,44,46,49,50,54,58], and 22 papers [23,25,27,29,32-36,38-41,44-46,49,50,54,55,58,59] provided no information on the blinding of participants and personnel. As data were often collected online, the detection bias in 16 papers [26,30,31,33-35,37,38,40,42,43,49,51,53,59,60] was determined to be low risk. Researchers in 21 studies [23,26-28,30-32,34,44,47-51,53-57,59,60] reported completeness of data. In addition, reporting bias was uncertain in most papers (n=26) [23,24,26,27,29,32,33,35-39,41-46,48, 50,51,54,55,58-60]. The overall quality of the included studies was moderate, and further high-quality studies are needed.

### Completeness of Reporting

**Figure 3:**
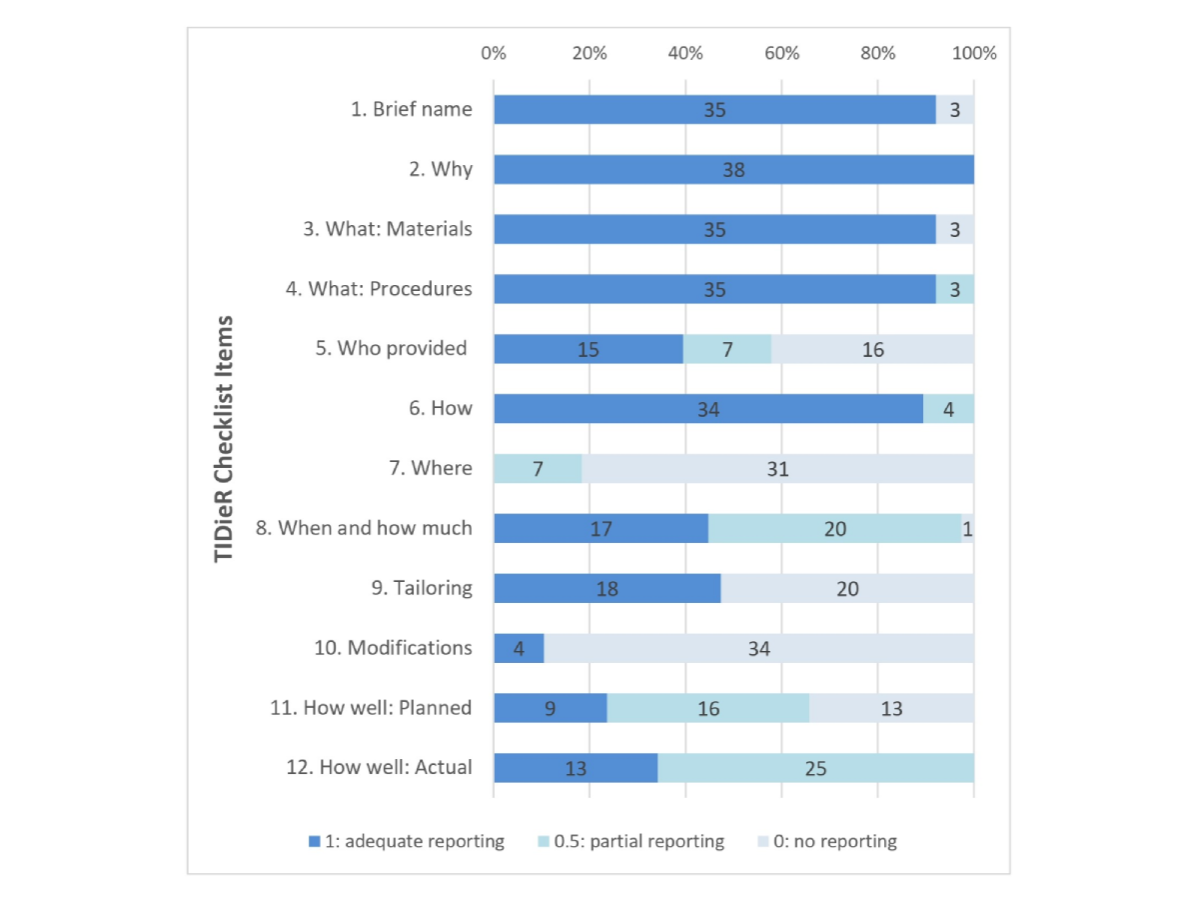
Distribution of studies with different scores in each item of the Template for Intervention Description and Replication-Telehealth checklist. Numbers in bars represent the number of interventions rated as 1 (adequate reporting), 0.5 (partial reporting), and 0 (no reporting). TIDieR-Telehealth: Template for Intervention Description and Replication-Telehealth.

The item scores for each trial are shown in Table 1 [23-60]. None of the papers described all the items on the TIDieR-Telehealth checklist. The completeness of the reporting score varied between 5 and 11.5, with a mean of 7.7. Figure 3 shows the distribution of studies with different scores in each item of the TIDieR-Telehealth checklist. Nearly all trials (n=35) reported the name, the use of physical or informational materials, and the procedures of the interventions (item 1, item 3, item 4). All trials described a rationale (item 2). More than half (n=22) of the included studies [23,25,26,29,30,35,37,38,41,42, 45-47,50-52,54,55,57-60] mentioned intervention providers (item 5), and 68% (15/22) of the studies [26,29,30,35,37,45-47,50,51,54,55,57-59] provided detailed information of the providers, including their expertise, background, and any specific training given. All trials described the mode of delivery (item 6), and 4 papers [26,39,53,54] did not mention whether the interventions were provided individually or in a group. Only 7 studies [29,32,34,35,45,56,57] mentioned the types of locations where the intervention occurred (item 7). The majority of the studies (37/38, 98%) mentioned the number of times the intervention was delivered, while fewer than half (17/37) [23,25,28-30,32,35,37,38, 41,42,44,50,55-57,60] reported the schedule and duration (item 8). Nearly half (n=18) of the studies [23,32,33,35,38,40-42,44-46,49-53,55,57] reported the tailoring of the interventions (item 9). The least reported item was item 10, with only 4 papers [25,28,35,48] mentioning modification of the intervention. Item 11, that is, strategies to improve fidelity/adherence, was reported in 25 (66%) of the 38 studies [23-27,31,32,34-38,40,45-48,51-55,57,59,60]. Only 13 (34%) studies [24,28,31,33,35-37,44-46,54,57,59] provided a complete description of the actual fidelity of the intervention delivery (item 12).

**Table 1:** Template for Intervention Description and Replication-Telehealth scores [23-60].

Author	Template for Intervention Description and Replication-Telehealth items													Final score
	1 (n=35)	2 (n=38)	3 (n=35)	4 (n=36.5)	5 (n=18.5)	6 (n=36)	7 (n=3.5)	8 (n=27)	9 (n=18)	10 (n=4)	11 (n=17)	12 (n=25.5)		
													
Au et al [23]	1	1	1	1	0.5	1	0	1	1	0	0.5	0.5	8.5	
Baruah et al [24]	1	1	1	1	0	1	0	0.5	0	0	0.5	1	7	
Berwig et al [25]	1	1	1	1	0.5	1	0	1	0	1	0.5	0.5	8.5	
Blom et al [26]	1	1	1	1	1	0.5	0	0.5	0	0	1	0.5	7.5	
Christie et al [27]	1	1	1	1	0	1	0	0.5	0	0	1	0.5	7	
Cristancho-Lacroix et al [28]	1	1	1	1	0	1	0	1	0	1	0	1	8	
De Stefano et al [29]	1	1	0	1	1	1	0.5	1	0	0	0	0.5	7	
Dichter et al [30]	1	1	1	1	1	1	0	1	0	0	0	0.5	7.5	
Duggleby et al [31]	1	1	1	1	0	1	0	0.5	0	0	1	1	7.5	
Eisdorfer et al [32]	1	1	1	1	0	1	0.5	1	1	0	0.5	0.5	8.5	
Finkel et al [33]	1	1	1	1	0	1	0	0.5	1	0	0	1	7.5	
Gaugler et al [34]	1	1	1	1	0	1	0.5	0.5	0	0	0.5	0.5	7	
Glueckauf et al [35]	1	1	1	1	1	1	0.5	1	1	1	1	1	11.5	
Gustafson et al [36]	1	1	1	1	0	1	0	0.5	0	0	0.5	1	7	
Han et al [37]	1	1	1	1	1	1	0	1	0	0	0.5	1	8.5	
Hepburn et al [38]	1	1	1	1	0.5	1	0	1	1	0	0.5	0.5	8.5	
Kajiyama et al [39]	1	1	1	1	0	0.5	0	0.5	0	0	0	0.5	5.5	
Kales et al [40]	0	1	1	1	0	1	0	0.5	1	0	0.5	0.5	6.5	
Karagiozi et al [41]	1	1	1	1	0.5	1	0	1	1	0	0	0.5	8	
Kwok et al [42]	1	1	1	1	0.5	1	0	1	1	0	0	0.5	8	
Mahoney et al [43]	1	1	1	1	0	1	0	0.5	0	0	0	0.5	6	
Martindale-Adams et al [44]	1	1	1	1	0	1	0	1	1	0	0	1	8	
Mavandadi et al [45]	1	1	1	1	1	1	0.5	0.5	1	0	0.5	1	9.5	
Meichsner et al [46]	1	1	1	1	1	1	0	0.5	1	0	1	1	9.5	
Meichsner et al [47]	1	1	1	1	1	1	0	0.5	0	0	0.5	0.5	7.5	
Moskowitz et al [48]	1	1	1	1	0	1	0	0.5	0	1	0.5	0.5	7.5	
Núñez-Naveira et al [49]	1	1	1	1	0	1	0	0.5	1	0	0	0.5	7	
Pérez et al [50]	0	1	1	1	1	1	0	1	1	0	0	0.5	7.5	
Schoenmakers et al [51]	0	1	0	0.5	1	1	0	0.5	1	0	0.5	0.5	6	
Su et al [52]	1	1	1	1	0.5	1	0	0.5	1	0	0.5	0.5	8	
Teles et al [53]	1	1	1	1	0	0.5	0	0.5	1	0	1	1	8	
Töpfer et al [54]	1	1	1	0.5	1	0.5	0	0.5	0	0	0.5	0.5	6.5	
Tremont et al [55]	1	1	1	1	1	1	0	1	1	0	1	0.5	9.5	
Williams et al [56]	1	1	1	1	0	1	0.5	1	0	0	0	0.5	7	
Wilz et al [57]	1	1	1	1	1	1	0.5	1	1	0	1	1	10.5	
Winter and Gitlin [58]	1	1	0	0.5	1	1	0	0	0	0	0	0.5	5	
Xu et al [59]	1	1	1	1	1	1	0	0.5	0	0	1	1	8.5	
Zarei et al [60]	1	1	1	1	0.5	1	0	1	0	0	0.5	0.5	7.5	

## Discussion

### Principal Findings

This review is a retrospective study evaluating the completeness of reporting on telehealth interventions for caregivers of people with dementia. The principal finding was that none of the telehealth interventions were reported in enough detail to satisfy every TIDieR-Telehealth checklist item, despite the checklist being published in 2022. This finding confirms the need for the introduction of the TIDieR-Telehealth checklist and suggests that there is a large shortfall in the reporting of information needed to accurately replicate telehealth interventions. In particular, the least frequently reported items were the ones referring to the type of location of the interventions (fully reported by no study), as well as the modifications (fully reported by 4 studies [25,28,35,48]), and fidelity assessment during the study (fully reported by 9 studies [26,27,31,35,46,53,55,57,59]). Importantly, these elements may be fundamental for enabling interventions to be adequately replicated, compared, and transferable in clinical practice [16].

Similar to our findings, a previous systematic review showed that the reporting of telehealth-delivered dietary interventions in chronic disease is inadequate, although this was assessed using the TIDieR checklist [61]. Given that the TIDieR-Telehealth checklist was published in 2022, there are currently no studies describing the completeness of telehealth interventions. However, some studies evaluated using the TIDieR checklist also indicated inadequate completeness of reporting across trials of weight management, cardiac rehabilitation, and manipulation and mobilization techniques [15,16,18]. Compared to those studies, the proportion of papers with complete descriptions of the intervention in our study was different. It might be that telehealth intervention had its unique evaluation standard: the TIDieR‑Telehealth checklist. Another explanation might be that our interpretation of the TIDieR‑Telehealth checklist and guide produced a more stringent evaluation than previous studies of interventions reporting quality, and we adopted a more detailed way of keeping the score.

Unlike other studies [18,62], the names of the included trials were not fully reported as required, because the TIDieR-Telehealth checklist requires that the name of the trial should include the word “telehealth,” which differs from the requirements in the TIDieR checklist. Most studies (>88%) reported intervention materials, procedures, and modes of delivery, which were similar to the findings of previous studies [15,18]. Information on intervention providers, such as category, experience, background, and any specific training given, was fully described in only 15 studies [26,29,30,35,37,45-47,50,51,54,55,57-59]. These descriptions are important for telehealth interventions because the delivery of telehealth interventions requires training to ensure the normativity of the study, and the expertise and disciplinary background of the provider might affect the outcome of a trial [18]. None of the trials fully described the location of the intervention, which may be related to the characteristic that telehealth is not limited by the location. However, the authors of the TIDieR checklist and guide emphasized that the location(s) of a trial might impact intervention feasibility and adherence. Nearly half of the studies fully reported the duration and number of interventions. Some interventions using remote monitoring systems or telehealth support programs were designed for participants to use when needed; therefore, the length and the number of interventions are not described. However, the arrangement of these interventions has a great influence on the effect of the intervention; therefore, the evaluation of the fidelity of intervention is particularly important. Tailoring, as an important part of the intervention, was mentioned in 18 trials [23,32,33,35,38,40-42,44-46,49-53,55,57]. The application parameters of technology are no longer predetermined but are constantly adjusted due to the feedback of the patients and the tailoring of the therapist to the responses of the individual patient [63]. Frequent shortcomings were items related to the reporting of modifications and the fidelity of interventions. In this study, only 4 trials (11%) [25,28,35,48] mentioned modifications in the interventions, which are essential for refining the intervention content and enhancing the replicability and the validity of the trial. Moreover, only 9 studies [26,27,31,35,46,53,55,57,59] fully described the methods for assessing and improving the fidelity of intervention. The assessment of fidelity remains a challenge in intervention trials, particularly in telehealth, where maintaining consistency can be difficult. Given that fidelity substantially affects treatment outcomes, this is an important factor to consider when interpreting study results [14,17].

### Limitation and Strengths

Different from other studies [15,61], our research employs a 3-point method, accounting for both fully and partially reported items. From these statistics, we can identify the missing parts in a report and urge that study to improve the completeness of that report. Moreover, one limitation of our study is that we did not compare the completeness of a report before and after the publication of the TIDieR‑Telehealth checklist, as this checklist has been published only recently and related studies were limited. More trials are needed to confirm the usefulness of this checklist.

### Conclusion

We found the reporting of interventions according to the TIDieR-Telehealth checklist to be inadequate in our sample of related trials. Fundamental details were often not described, affecting research validity and reproducibility. Future RCTs on telehealth interventions for dementia should adhere to standardized reporting. It is necessary to consider other mechanisms to improve TIDieR adherence or to find other solutions to improve intervention reporting. Meanwhile, telehealth interventions, as an intervention method in the new era, have brought many benefits to patients and caregivers but also posed challenges to intervention providers. Therefore, the next step in the implementation of telehealth interventions is how to properly utilize the benefits of telehealth to maximize its value.

## Appendix

Multimedia Appendix 1 Search strategy.

Multimedia Appendix 2 PRISMA (Preferred Reporting Items for Systematic Reviews and Meta-Analyses).

Multimedia Appendix 3 Characteristics of the eligible randomized controlled trials [26-63].

## Abbreviations

CONSORT: Consolidated Standards of Reporting Trials
RCT: randomized controlled trial
TIDieR: Template for Intervention Description and Replication
TIDieR-Telehealth: Template for Intervention Description and Replication-Telehealth
