# Supplementary material for: Completeness of Telehealth Interventions Reporting in Randomized Controlled Trials for Caregivers of People With Dementia: Systematic Review
Source: J Med Internet Res. 2025 Jan 20;27:e53737. doi: 10.2196/53737 (PMC11791455; doi:10.2196/53737)
Supplement: Multimedia Appendix 1 [file jmir_v27i1e53737_app1.docx]

**Search Strategy**

**PubMed**

#1 Search: "Caregivers"[Mesh]

#2 Search: ("Dementia"[Mesh]) OR ("Alzheimer Disease"[Mesh])

#3 Search: ((("Telemedicine"[Mesh]) OR ("Internet"[Mesh])) OR ("Telephone"[Mesh])) OR ("Software"[Mesh])

#4 Search: (((caregiver*[Title/Abstract]) OR ("care giver*"[Title/Abstract])) OR (carer*[Title/Abstract])) OR (caretaker*[Title/Abstract])

#5 Search: (((dementia*[Title/Abstract]) OR (alzheimer*[Title/Abstract])) OR (cognit* impair*[Title/Abstract])) OR (AD[Title/Abstract])

#6 Search: ((((((((((((telehealth[Title/Abstract]) OR (mHealth[Title/Abstract])) OR (eHealth[Title/Abstract])) OR ("Mobile Health"[Title/Abstract])) OR (remote[Title/Abstract])) OR (online[Title/Abstract])) OR (web[Title/Abstract])) OR (phone[Title/Abstract])) OR (ipad[Title/Abstract])) OR (computer[Title/Abstract])) OR (app[Title/Abstract])) OR (application[Title/Abstract])) OR (video*[Title/Abstract])

#7 Search: #1 OR #4

#8 Search: #2 OR #5

#9 Search: #3 OR #6

#10 Search: "randomized controlled trial"[pt] OR "controlled clinical trial"[pt] OR randomized[tiab] OR placebo[tiab] OR "drug therapy"[sh] OR randomly[tiab] OR trial[tiab] OR groups[tiab]

#11 Search: #7 AND #8 AND #9 AND #10

**Cochrane**

#1 MeSH descriptor: [Caregivers] explode all trees

#2 (caregiver* OR “care giver*” OR carer* OR caretaker*):ti,ab,kw

#3 #1 OR #2

#4 MeSH descriptor: [Dementia] explode all trees

#5 MeSH descriptor: [Alzheimer Disease] explode all trees

#6 (dementia* OR alzheimer* OR “cognit* impair*” OR AD):ti,ab,kw (Word variations have been searched)

#7 #4 OR #5 OR #6

#8 MeSH descriptor: [Telemedicine] explode all trees

#9 MeSH descriptor: [Software] explode all trees

#10 MeSH descriptor: [Telephone] explode all trees

#11 MeSH descriptor: [Internet] explode all trees

#12 (telehealth OR mHealth OR eHealth OR “Mobile Health” OR remote OR online OR web OR phone OR ipad OR computer OR app OR application OR video*):ti,ab,kw (Word variations have been searched)

#13 #8 OR #9 OR #10 OR #11 OR #12

#14 #3 AND #7 AND #13

**CINAHL**

S1 (MH "Caregivers")

S2 (MH "Dementia") OR (MH "Alzheimer's Disease")

S3 (MH "Internet") OR (MH "Telemedicine") OR (MH "Software") OR (MH "Telephone")

S4 TI ( caregiver* OR carer* OR "care giver*" OR caretaker* ) OR AB ( caregiver* OR carer* OR "care giver*" OR caretaker* )

S5 TI ( dementia* OR alzheimer* OR "cognit* impair*" OR AD ) OR AB ( dementia* OR alzheimer* OR "cognit* impair*" OR AD )

S6 TI ( video* OR phone OR ipad OR computer OR app OR application OR mHealth OR eHealth OR telehealth OR "Mobile Health" OR remote OR online OR web ) OR AB ( video* OR phone OR iPad OR computer OR app OR application OR mHealth OR eHealth OR telehealth OR "Mobile Health" OR remote OR online OR web )

S7 TI ( "randomized controlled trial" OR "controlled clinical trial" OR randomized OR randomly ) OR AB ( "randomized controlled trial" OR "controlled clinical trial" OR randomized OR randomly )

S8 S1 OR S4

S9 S2 OR S5

S10 S3 OR S6

S11 S7 AND S8 AND S9 AND S10

**Web of Science**

1: (((TS=(“randomized controlled trial”)) OR TS=(“controlled clinical trial”)) OR TS=(randomized)) OR TS=(randomly)

2: TS=(dementia* OR “Alzheimer disease” OR alzheimer* OR “cognit* impair*” OR AD)

3: TS=(caregiver* OR “care giver*” OR carer* OR caretaker*)

4: TS=(Telemedicine OR Software OR Internet* OR video* OR phone OR iPad OR computer OR app OR application OR mHealth OR eHealth OR telehealth OR “Mobile Health” OR remote OR online OR web OR telephone)

5: #1 AND #2 AND #3 AND #4

**Embase**

#1 'caregiver'/exp

#2 'dementia'/exp OR 'alzheimer disease'/exp

#3 'telemedicine'/exp OR 'internet'/exp OR 'telephone'/exp OR 'software'/exp

#4 dementia*:ab,ti OR alzheimer*:ab,ti OR 'cognit* impair*':ab,ti OR ad:ab,ti

#5 caregiver*:ab,ti OR 'care giver*':ab,ti OR carer*:ab,ti OR caretaker*:ab,ti

#6 video*:ab,ti OR phone:ab,ti OR ipad:ab,ti OR computer:ab,ti OR app:ab,ti OR application:ab,ti OR mhealth:ab,ti OR ehealth:ab,ti OR telehealth:ab,ti OR 'mobile health':ab,ti OR remote:ab,ti OR online:ab,ti OR web:ab,ti

#7 'randomized controlled trial'/exp OR 'controlled clinical trial'/exp OR randomized:ti,ab OR placebo:ti,ab OR 'drug therapy':lnk OR randomly:ti,ab OR trial:ti,ab OR groups:ti,ab

#8 #1 OR #5

#9 #3 OR #6

#10 #2 OR #4

#11 #7 AND #8 AND #9 AND #10

CNKI, CBM, WanFang, VIP

#1 痴呆 OR 阿尔茨海默 OR 失智 OR 认知症

#2 照顾者 OR 照料者 OR 照护者 OR 护理人员

#3 远程 OR 互联网 OR 网络 OR 电话 OR 在线 OR 移动 OR 手机 OR 电脑 OR app OR 微信 OR 视频

#4 #1 AND #2 AND #3
