# Supplementary material for: Completeness of Telehealth Interventions Reporting in Randomized Controlled Trials for Caregivers of People With Dementia: Systematic Review
Source: J Med Internet Res. 2025 Jan 20;27:e53737. doi: 10.2196/53737 (PMC11791455; doi:10.2196/53737)
Supplement: Multimedia Appendix 3 [file jmir_v27i1e53737_app3.docx]

**Table S1.** Study characteristics [26-63].

| **Author** | **Year** | **Country** | **Population (Females)**  **(IG/CG)** | **Age (years), mean(SD)** | **Severity of dementia** | **Interventions** | **Administrator** | **Delivery** | **Duration (weeks)** | **Comparison** | **Primary Outcomes** | **Assessment time** | **Trial registry [yes/no]** |
| --- | --- | --- | --- | --- | --- | --- | --- | --- | --- | --- | --- | --- | --- |
| Au et al [23] | 2014 | China | 30/30 (22/24) | 58.1(12.4)/ 55.1(11.3) | Mild to severe | Psychoeducational intervention | Psychogeriatric team | Telephone | 4 | Usual care | Depression,  Self-efficacy | Baseline,  1 month,  2 months | No |
| Baruah et al [24] | 2021 | India | 74/77 (34/36) | 46.5(14.1)/ 42.2(11.9) | Mild to severe | Training and support program | NR | Internet (web) | 12 | Usual care | Burden,  Depression | Baseline,  3 months | Yes |
| Berwig et al [25] | 2022 | Germany | 107/114 (NR) | NR | NR | Aftercare group sessions | Social worker | Telephone | 24 | Usual care | Social participation | Baseline, 3 weeks, 6 months, 12 months | Yes |
| Blom et al [26] | 2015 | Netherlands | 149/96 (104/66) | 61.5(11.9)/ 60.8(13.1) | Mild | Innovative guided self-help course | Psychologist | Internet | 24 | Usual care | Depression | Baseline, 6 months | Yes |
| Christie et al [27] | 2022 | Netherlands | 48/48 (31/34) | 58.1(11.8)/  55.7(13.6) | NR | Social support intervention | NR | Internet (web+app) | 16 | Wait-list | Sence of competence,  Social support | Baseline,  8 weeks,  16 weeks | Yes |
| Cristancho-Lacroix et al [28] | 2015 | France | 25/24 (16/16) | 64.2(10.3)/ 59.0(12.4) | Mild to severe | Psychoeducational intervention | NR | Internet (web) | 12 | Usual care | Stress | Baseline,  3 months, 6 months | Yes |
| De Stefano et al [29] | 2022 | Italy | 10/10 (NR) | 49(14.9)/ 57.7(7.7) | Mild to severe | Psychoeducational intervention | Psychologist/ psychotherapist | Telephone | 4 | Usual care | Burden,  Anxiety,  Depression,  Impact of event,  Caregiver need | Baseline,  5 weeks,  7 months | No |
| Dichter et al [30] | 2020 | Germany | 19/19 16/16 | 67.4(8.1)/ 64.1(10.6) | NR | Social support intervention | Psychologist | Telephone | 6 | Usual care | The psychological health related quality of life | Baseline, 3 months | Yes |
| Duggleby et al [31] | 2018 | Canada | 101/98 (79/82) | 63.4(12.2)/ 63.9(11.1) | NR | Support intervention | NR | Internet (web) | 12 | Usual care | Health | Baseline,  1 month, 3 months, 6 months | Yes |
| Eisdorfer et al [32] | 2003 | USA | 225 (NR) | NR | Mild to severe | Family therapy  Technology-based intervention | NR | Computer–Telephone Integration System | 48 | Minimal support | Depression | Baseline,  6 months, 18 months | No |
| Finkel et al [33] | 2007 | USA | 23/23 (NR) | NR | NR | Communications technology intervention | Clinical social worker | Computer- Telephone Integration  System | 24 | Usual care | Depression | Baseline, 6 months | No |
| Gaugler et al 34] | 2021 | USA | 88/91 (68/73) | 62.4 (10.8)/ 63.0 (12.5) | NR | Remote activity monitoring | NR | Remote activity monitoring system | 72 | Attention control | Self-efficacy,  Sence of competence,  Distress | Baseline, 18 months | Yes |
| Glueckauf et al [35] | 2012 | USA | 7/7 (NR) | NR | NR | Cognitive-behavioral therapy | Counselor | Telephone | 12 | Face-to-face | Burden,  Social support,  Health,  Depression | Baseline, 12 weeks | No |
| Gustafson et al [36] | 2019 | USA | 16/15 (11/8) | NR | Mild to severe | Support intervention | NR | Internet (web) | 24 | Usual care | Burden,  Family conflict,  Satisfaction with decisions,  Loneliness,  Anxiety,  Depression,  Coping competence | Baseline,  2 months,  4 months,  6 months | No |
| Han et al [37] | 2023 | USA | 9/10 (9/10) | 54.6 | NR | Videoconferencing acceptance and commitment therapy | Counselor | Internet (videoconferencing) | 8 | Usual care | Depressive,  Anxiety,  Stress,  Burden,  Grief,  Guilt,  Psychological quality of life,  Psychological flexibility,  Cognitive fusion,  Subjective experience,  Self-compassion | Baseline, 8 weeks, 12 weeks | Yes |
| Hepburn et al [38] | 2022 | USA | 96/54 (72/39) | 66.0(10.9)/ 63.7(10.7) | NR | Psychoeducational intervention | NR | Internet | 6 | Wait-list | Burden,  Depression,  Anxiety,  Stress,  Caregiver mastery,  Behavioral and psychological symptoms | Baseline,  3 months,  6 months,  9 months,  12 months | No |
| Kajiyama et al [39] | 2013 | USA | 75/75 (125) | NR | NR | Stress management program | NR | Internet | 12 | Usual care | Stress | Baseline,  3 months | No |
| Kales et al [40] | 2018 | USA | 27/30 (19/24) | 65.5(11.8) / 66.2(15.9) | NR | Support intervention | NR | Internet (web) | 4 | Wait-list | Distress,  Confidence | Baseline, 1 month | Yes |
| Karagiozi et al [41] | 2022 | Greece | 27/44 (NR) | 42.6(12.6)/ 45.8(9.1) | Moderate | Psychoeducational intervention | Psychologist | Internet (video conferencing) | 16 | Usual care | Burden,  Depression,  Anxiety | Baseline, 4 months | No |
| Kwok et al [42] | 2013 | China | 18/20 (13/14) | NR | Moderate to severe | Psychoeducational intervention | Social worker | Telephone | 12 | Usual care | Burden,  Self-efficacy | Baseline,  3 months | No |
| Mahoney et al [43] | 2003 | USA | 49/51 (40/38) | 61.4(13.6)/ 63.7(11.8) | NR | Interactive voice response intervention | NR | Computer-Telephone Integration System | 48 | Usual care | Depression,  Anxiety,  Bother | Baseline,  6 months,  12 months,  18 months | No |
| Martindale-Adams et al [44] | 2013 | USA | 77/77 (63/66) | 66.2(12.3)/ 65.0(12.6) | Mild to severe | Support group intervention | NR | Telephone | 48 | Usual care | Burden,  Depression,  Health,  Self-care,  General well-being,  Frequency and bother of dementia behaviors | Baseline,  6 months, 12 months | No |
| Mavandadi et al [45] | 2017 | USA | 38/37 (36/37) | 71.97 (10.92)/ 67.94 (12.24) | NR | Collaborative care management program | Nurse,  Clinician | Telephone | 12 | Usual care | Frequency and severity of dementia-related symptom,  Distress | Baseline,  3 months, 6 months | No |
| Meichsner et al [46] | 2018 | Germany | 19/18 (14/15) | 63.0(9.4)/ 61.1(10.1) | Moderate to severe | Cognitive-behavioral intervention | Psychologist | Internet | 8 | Wait-list | Depression,  Grief,  Utilization of resources,  Burden | Baseline,  8 weeks, 5 months | No |
| Meichsner et al [47] | 2019 | Germany | 139/134 (220) | 64.10(11.04) | Mild to severe | Cognitive-behavioral intervention | Clinical psychologist | Telephone | 24 | Wait-list | Quality of life | Baseline,  6 months, 12 months | Yes |
| Moskowitz et al [48] | 2019 | USA | 86/84 (73/70) | 63.0(9.4)/ 62.7(10.1) | Mild to moderate | Positive emotion regulation intervention | NR | Internet | 6 | Wait-list | Positive and negative emotion | Baseline,  6 weeks,  10 weeks,  18 weeks,  30 weeks | Yes |
| Núñez-Naveira et al [49] | 2016 | Spain | 30/31 (21/18) | NR | Moderate to severe | Education programs,  Ambient assisted living intervention | NR | Internet (app) | 12 | Usual care | Depression,  Competence,  Satisfaction, | Baseline,  3 months | No |
| Pérez et al [50] | 2022 | Spain | 39/35 (66) | 37(9.13) | NR | Mindfulness-Based Intervention | Nurse,  Psychologist | Internet | 6 | Wait-list | Professional quality of life | Baseline,  6 weeks, 18 weeks | No |
| Schoenmakers et al [51] | 2010 | Belgium | 32/27 (25/20) | 64.4(12.9) /62.3(15.1) | NR | Support | Primary care professional, General practitioner | Telephone | 48 | Usual care | Depression | Baseline,  6 months, 12 months | No |
| Su et al [52] | 2021 | China | 70/70 (69/69) | 47.3(7.1) /48.4(8.7) | NR | Mentoring support | Home care service supervisor | Internet (app) | 12 | Usual care | Knowledge,  Attitude,  Competence | Baseline,  12 weeks,  24 weeks | Yes |
| Teles et al [53] | 2022 | Portugal | 21/21 (17/16) | 49(12.1)/ 58.1(12.5) | NR | Training and support program | NR | Internet (web) | 12 | Usual care | Burden,  Depression,  Anxiety,  Subjective quality of life,  Self-efficacy | Baseline,  3 months,  6 months | Yes |
| Töpfer et al [54] | 2023 | Germany | 139/49 (112/38) | 63.91 (11.47) /62.24 (11.36) | Mild to severe | Cognitive-behavioral intervention | Clinical psychologist | Telephone | 24 | Face-to-face | Depression,  Burden | Baseline,  6 months, 12 months | Yes |
| Tremont et al [55] | 2008 | USA | 16/17 (NR) | 65.8(13.7)/ 61.0(9.6) | Mild to moderate | Psychosocial intervention | Trained therapist | Telephone | 48 | Usual care | Burden,  Depression,  Reaction to memory and behavior problems | Baseline,  12 months | No |
| Williams et al [56] | 2010 | USA | 59/57 (44/46) | 62.1(13.6) /59.0(12.8) | NR | Coping skills intervention | NR | Internet | 5 | Wait-list | Stress,  Anxiety,  Anger,  Depression,  Hostility,  Self-efficacy,  Sleep | Baseline, 7 weeks,  3 months, 6 months | Yes |
| Wilz et al [57] | 2018 | Germany | 139/134 (220) | 64.2(11.0) | Mild to severe | Cognitive-behavioral intervention | Psychotherapist | Telephone | 24 | Usual care | Depression,  Emotional well-being,  Health | Baseline,  6 months,  12 months | Yes |
| Winter and Gitlin [58] | 2006 | USA | 58/45 (58/45) | 68.7(9.3)/ 64.0(8.2) | NR | Support group intervention | Social worker | Telephone | 24 | Usual care | Burden,  Depression,  Gains | Baseline,  6 months | No |
| Xu et al [59] | 2022 | China | 35/36 (27/26) | 54.71(11.19)/ 53.44 (10.83) | NR | Behavioral activation | Nursing doctoral researcher | Telephone | 8 | Usual care | Sleep quality,  Depression | Baseline. 8 weeks, | No |
| Zarei et al [60] | 2022 | Canada | 14/12 (13/10) | 58(11)/ 63(15) | NR | Mindfulness-based cognitive therapy | Mental health clinician | Internet (videoconference program) | 8 | Usual care | Stress,  Depression,  Anxiety,  Coping,  Self-Compassion | Baseline,  8 weeks,  12 weeks | No |
